# Will Web Surveys Ever Become Part of Mainstream Research?

**DOI:** 10.2196/jmir.6.3.e31

**Published:** 2004-09-23

**Authors:** Matthias Schonlau

**Affiliations:** ^1^RAND CorporationSanta Monica CAUSA

This issue contains two interesting papers on Web survey methodology, which reach different conclusions about the potential of Web surveys. Particular attention is directed to relative response rates. A high response rate is commonly taken as an indicator of survey validity.

Leece et al used systematic sampling to assign one half of a list of orthopedic surgeons to a Web survey and the other half to a mail survey [1]. They observed that the Web survey produced a significantly lower response rate than the mail survey, and cautioned, “Researchers should not assume that the widespread availability and potential ease of Internet-based surveys will translate into higher response rates.” In contrast, Ritter et al, who recruited participants from the Internet and randomly assigned them either to a mail survey or to a Web survey, observed different results [2]. They found that participation was at least as good as if not better among the Web survey group than among those receiving questionnaires by mail.  In addition the investigators found that the responses to 16 health-related questions did not differ significantly between the two study groups.

The different findings can be explained by the respective recruiting strategies. Ritter et al recruited participants over the Internet. Clearly, respondents recruited on the Web are more likely to respond to a Web survey than the general population. The finding is nonetheless interesting because it is not obvious that the response rate to a Web survey would be higher than to a mail survey even among Internet-savvy respondents. A Web survey typically achieves a higher response rate when respondents are contacted by e-mail rather than by mail [3]. Analogously, a mail survey typically achieves a higher response rate when respondents are contacted by mail rather than by e-mail. It is possible that recruiting respondents on the Web also reduces the response rate of a mail survey because the recruiting mode is different from the response mode.

Both Ritter et al and Leece et al survey special rather than general populations [1,2]. Ritter et al recruit respondents from the Internet [2]. Leece et al have a master list of orthopedic surgeons [1]. They also have e-mail addresses for 79% (all but 45 respondents) of the respondents in the Web survey arm. A much greater challenge would be to conduct a Web survey of a general population for which no master list of e-mail addresses is readily available. One approach, contacting respondents by mail and encouraging response by Web with a mail fallback option, is discussed in Schonlau et al [4]. This approach is not very practical because the second response mode requires additional resources and slows the survey down.

Ritter et al's survey and most Web surveys are conducted with convenience samples rather than with random samples [2]. In a convenience sample participants are selected, in part or in whole, at the convenience of the researcher. In a random sample the researcher ensures that each member of that population has a known probability (for example, equal probability) of being selected. For example, a sample of respondents recruited from newsgroup postings is a convenience sample for most populations of interest. Eysenbach and Wyatt note, “In 'open' web-based surveys, selection bias occurs … through self-selection of participants, …” [5]. Such selection bias implies a convenience sample because the probability of selection is unknown.

Whether Web surveys will develop into mainstream survey research tools depends on the possibility of drawing inferences from convenience samples. Conventional survey sampling wisdom holds that inferences cannot be drawn from convenience samples, thereby negating their use—with the possible exception of pilot studies. Still, convenience samples can be used to conduct experiments within that sample. Ritter et al have shown this with a nice properly-randomized experiment within a convenience sample; whether the larger sample is representative is secondary [2]. Ritter et al's finding would not hold for people without access to the Internet [2]. Other experiments can be conducted with a single convenience sample, including testing of response order effects (in visual response modes the first answer choice tends to be chosen more often) and of anchoring effects (the answer choice may be affected by the context, including what was asked in previous questions). Vignettes and factorial experiments could be inserted in Web surveys based on convenience samples. These are exciting research possibilities.

The possibility of drawing inferences from convenience samples is a contentious issue among survey researchers. The excitement needs to be tempered with rational skepticism.

Health service and biostatistical researchers have traditionally drawn conclusions from observational studies. The purpose of the ubiquitous “Table 1” of epidemiological cohort studies which displays demographical and other information on both experimental and control groups is to argue that experimental and control groups are not different with respect to important confounding variables, such as age and education. Therefore observed risk or outcome differences between the groups are indeed due to the exposure to the intervention (or treatment) and not to observed confounding factors. In a randomized study, the experimental design should “automatically” balance the covariates. For example, it is unlikely that participants in the exposed (intervention or treatment) group are significantly older than in the non-exposed (control) group. In a non-randomized study, such systematic differences are likely to occur due to selection bias. If in a non-randomized study one can show that the covariates are balanced, then there is little reason to distrust regression results or other inferences based on observational data.

Rubin's framework for causal inference goes further ensuring that the covariates in Table 1 are balanced [6]. Propensity scores are constructed from logistic regression on baseline variables that are thought to capture the difference between Web respondents and the general population. The propensity scores can be used to construct subclasses in which covariates are approximately balanced. One very important assumption is that no important unobserved variables affect treatment assignment. Rubin's approach is widely accepted.

Harris Interactive, a commercial Web survey company, has adapted Rubin's approach for drawing inferences from Web surveys [7]. Assignment to treatment or control corresponds to “assignment” of a respondent to a random or a convenience sample. Capturing the selection mechanism that distinguishes a random sample from the convenience sample allows for adjustment for it. While the selection approach of Harris Interactive is theoretically sound, the challenge is to ask the right questions to capture the difference between the online and offline populations. I am involved in a study which explores the feasibility of moving a portion of the Health and Retirement Survey (HRS), a large-scale US panel survey, onto the Internet in future survey waves. I have recently applied the propensity scoring approach to the HRS with early encouraging results [8].

Will inferences drawn from convenience samples achieve the rigor required by mainstream research? I am hopeful of this possibility. In the past researchers have rejected the possibility of drawing inferences from mail surveys because they were self-administered. Currently mail surveys are certainly considered “mainstream”. The possibility of inference based on convenience samples is one of several exciting research opportunities in Web survey research. Leece et al and Ritter et al have stimulated us to further consideration of the expanding research frontier [1,2].
